# The calcified Sphenomandibular ligament: case report, surgical management and literature review

**DOI:** 10.3389/froh.2025.1581583

**Published:** 2025-04-24

**Authors:** Shareef Araidy, George Batshon, Roman Mirochnik, Imad Abu El-Naaj

**Affiliations:** Department of Oral and Maxillofacial Surgery, Tzafon Medical Center, Affiliated with Azrieli Faculty of Medicine, Bar Ilan University, Ramat Gan, Israel

**Keywords:** trismus, OMFS, sphenomandibular ligament, sphenomandibular ligament calcification, dental complication, dental complications, pterygomandibular space

## Abstract

Calcification of the Sphenomandibular ligament (SML) is an extremely rare condition, with only three previously documented cases worldwide. This report presents the fourth known case of SML calcification in a 32-year-old male patient with a 17-year history of progressive trismus. Following a dental procedure involving a mandibular nerve block, the patient gradually developed limited mouth opening, which was resistant to conservative treatments. Imaging revealed calcification of the SML, which was confirmed intraoperatively. Surgical excision led to a significant improvement in mandibular function. This report discusses clinical presentation, diagnostic workup, surgical management, and a comprehensive literature review, emphasizing the importance of considering rare ligamentous pathologies in cases of persistent trismus.

## Introduction

The temporomandibular joint (TMJ) is a complex synovial joint that facilitates mandibular movement, enabling essential functions such as mastication, speech, and facial expression (1–3). The stability and function of the TMJ are supported by a network of ligaments, including the Sphenomandibular Ligament (SML), which is one of the accessory ligaments of the joint (1–3). This ligament plays a role in limiting excessive mandibular movements and stabilizing the joint during function (1–4).

To better understand the clinical significance of SML calcification, it is essential to review the anatomy of the region. The SML is a thin, fibrous band that extends from the spine of the sphenoid bone to the lingula of the mandible (4–6). It lies medial to the lateral pterygoid muscle and lateral to the medial pterygoid muscle, forming part of the infratemporal fossa (4–6). The ligament is in close proximity to the mandibular nerve and inferior alveolar vessels, which pass through the mandibular foramen (4–6). This anatomical relationship underscores the importance of careful surgical planning to avoid iatrogenic injury to these structures (5, 6).

Due to its anatomical characteristics, SML pathology is rare (4, 5). However, when pathology does occur, it can lead to significant functional impairments, such as trismus (limited mouth opening) (6, 7), which can severely impact a patient's quality of life (9, 10). Calcification of the SML is an exceedingly rare phenomenon, with only three documented cases in the medical literature to date (11, 12).

Calcification of ligaments and soft tissues is typically associated with degenerative processes, trauma, or metabolic disorders (13, 14). However, in the case of the SML, the etiology of calcification remains poorly understood due to the rarity of reported cases (11, 12). The first documented case, reported in 2014, involved a 46-year-old male from Bahrain who presented with progressive trismus and was found to have unilateral calcification of the SML (11). The second case, reported in 2023, described a 7-year-old boy from Yemen with severe trismus due to SML calcification. Both cases required surgical intervention to restore mandibular function (12). The third case recently diagnosed in 2025, involved an 8-year-old girl from Belgium who presented with a rapidly progressive mouth-opening limitation. The patient did not undergo surgical intervention; instead her condition resolved with maxillofacial physiotherapy (15).

This report presents the fourth documented case worldwide of SML calcification, involving a 32-year-old male who developed progressive trismus following a routine dental procedure. The patient's clinical presentation, diagnostic workup, and surgical management are described in detail, along with a comprehensive review of the existing literature. This case emphasizes the necessity of considering rare ligamentous pathologies in the differential diagnosis of trismus, particularly when conventional causes such as muscular fibrosis or TMJ dysfunction have been ruled out.

## Case presentation

A 32-year-old male with no significant medical history presented with a 17-year history of progressive trismus. The onset of symptoms occurred in 2007 following a routine dental procedure, during which the patient received a right mandibular nerve block injection for a restorative treatment. Although no immediate complications were reported, the patient gradually developed increasing difficulty in opening his mouth. Notably, the trismus was not associated with pain, clicking, or locking of the temporomandibular joint (TMJ), which are commonly observed in TMJ disorders (7, 8, 16–18).

Over the years, the patient sought multiple consultations with dental and medical professionals. Initial evaluations attributed the trismus to muscular fibrosis or TMJ dysfunction, both of which are more common causes of restricted mouth opening (7, 8, 16–18). Conservative management strategies, including physiotherapy and the use of oral appliances, were employed; however, these interventions failed to yield significant improvement in the patient's condition. This lack of response to conventional therapies prompted further investigation into potential rare or atypical causes of trismus.

## Clinical examination

On clinical examination, the patient exhibited a maximal interincisal opening (MIO) of 18 mm, which is significantly reduced compared to the normal range of 40–55 mm (7). No deviation of the mandible was observed during mouth opening or closing, and there was no evidence of tenderness, crepitus, clicking, or locking of the TMJ. Palpation of the masticatory muscles, including the temporalis, masseter, and medial pterygoid muscles, revealed no tenderness or signs of muscular pathology.

Routine blood tests, including complete blood count (CBC), chemistry panel, and coagulation studies, were within normal limits, with no indicators of systemic inflammation, infection, or metabolic abnormalities. The absence of systemic findings further supported a localized, structural etiology for the patient's trismus.

## Imaging studies

**Figure 1:**
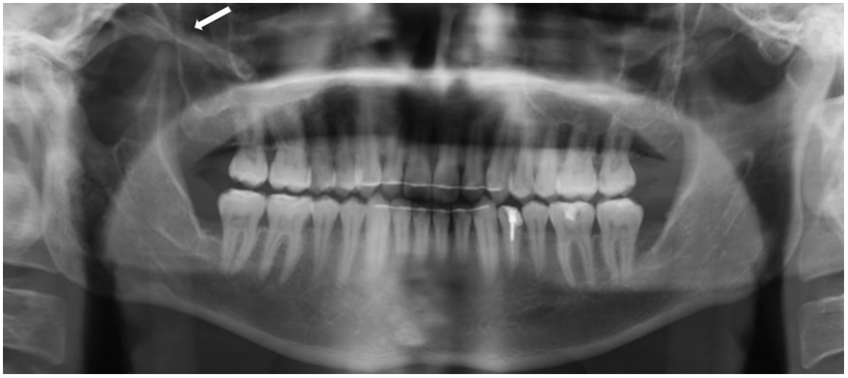
OPG imaging showing a radiopaque, elongated structure adjacent to the right coronoid process of the mandibular ramus (white arrow).

**Figure 2:**
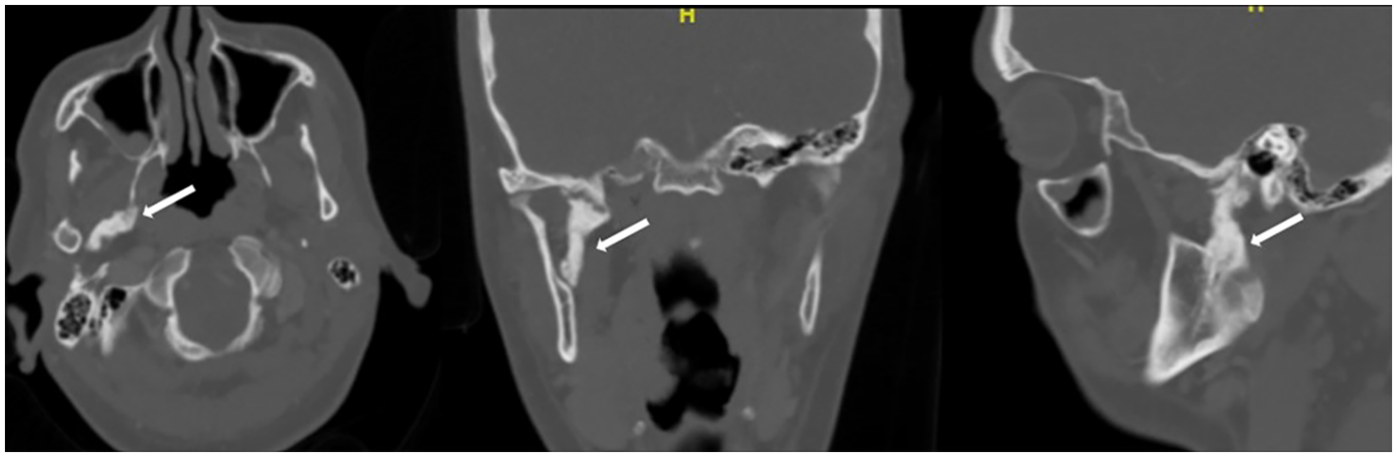
CT scan reconstructed in axial, coronal, and sagittal planes, confirmed the presence of a dense, linear calcification extending from the spine of the sphenoid bone to the lingula of the mandible (white arrow).

Initial imaging with a panoramic radiograph (OPG) revealed a radiopaque, elongated structure adjacent to the right coronoid process of the mandibular ramus (Figure 1). This finding prompted further investigation with computed tomography (CT), which demonstrated an elongated, calcified structure along the expected anatomical course of the right SML. The CT scan, reconstructed in axial, coronal, and sagittal planes, confirmed the presence of a dense, linear calcification extending from the spine of the sphenoid bone to the lingula of the mandible (Figure 2). The imaging findings were consistent with calcification of the SML. The calcified ligament appeared as a well-defined, hyperdense structure on CT, with no evidence of adjacent soft tissue inflammation or bony erosion.

## Diagnosis

The patient's history of symptom onset following a dental procedure, coupled with the absence of pain, clicking, or locking of the TMJ, ruled out more common causes of trismus, such as TMJ dysfunction or myofascial pain disorders. Based on the patient's 17-year history of progressive trismus, combined with the clinical examination and imaging findings, a definitive diagnosis of calcification of the SML was established as the primary cause of his limited mouth opening. Both OPG and CT scan allowed for the exclusion of other potential etiologies, such as tumors, infections, or TMJ ankylosis.

## Surgical management

**Figure 3:**
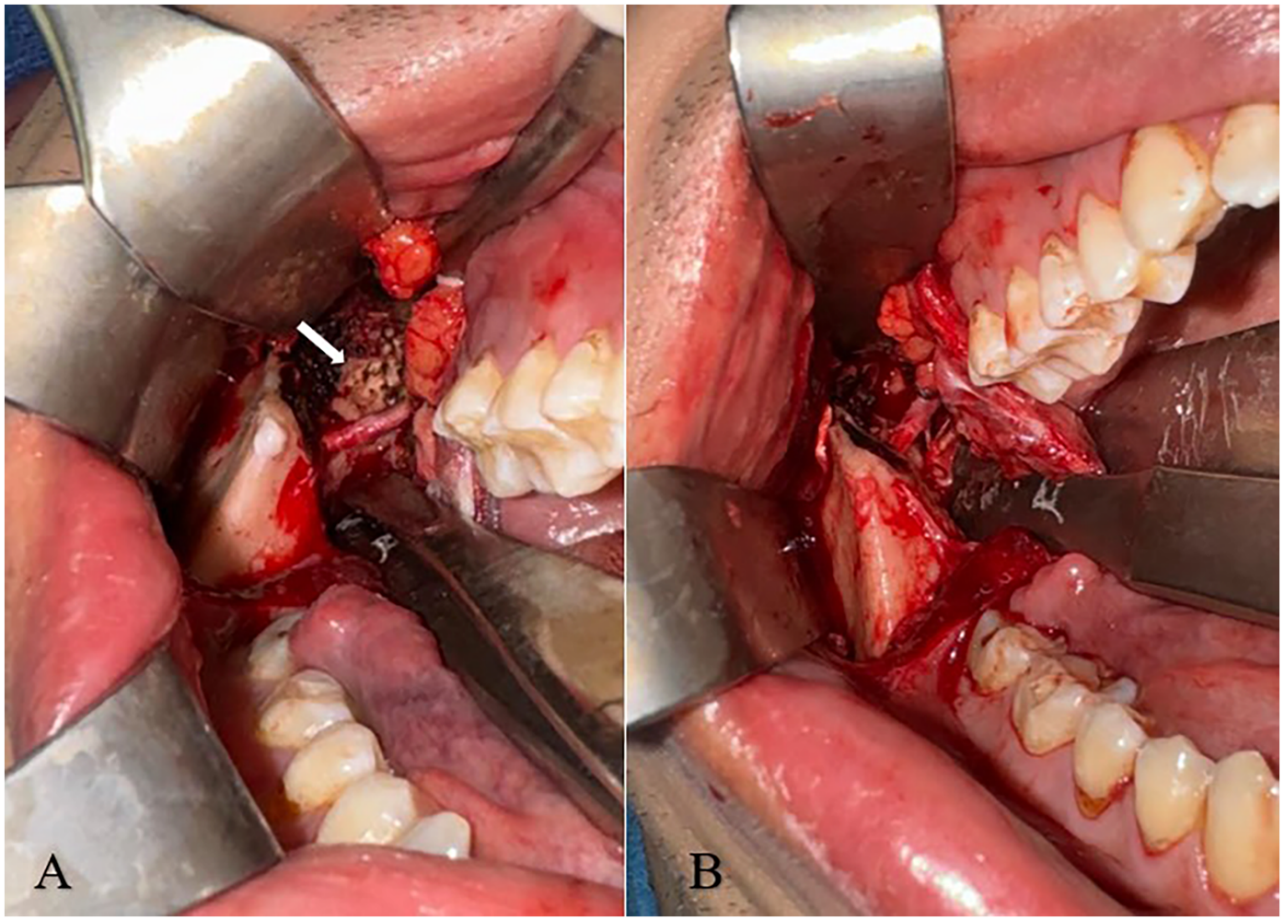
**(A,B) (A-left)** intra-operative image exhibiting the fully exposed SML (white arrow) following coronoidectomy. **(B-right)** Following excision of the SML.

The patient was offered surgical intervention to address the calcified SML. Under general anesthesia, a transoral approach was used to access the mandibular lingula. A mucosal incision was made from the lateral aspect of the retromolar fossa on the buccal mucosa, extending to the second molar, and further anteriorly along the vertical ramus to ensure adequate exposure of the ascending mandibular ramus. Dissection was carried superiorly until the sigmoid notch was reached. During the procedure, particular attention was given to identifying and protecting the neurovascular bundle entering the lingual foramen inferiorly. A Coronoidectomy was then performed solely to facilitate adequate retraction and visualization of the SML; no coronoid process abnormalities contributing to trismus were identified in this patient. The calcified SML was clearly identified, fully exposed (Figure 3A), and completely excised (Figure 3B). Remnants of the ligament at its origin and insertion sites were meticulously removed using a surgical file, while a small calcification at the lingula was left undisturbed to avoid unnecessary trauma to the surrounding structures. Hemostasis was achieved using diathermy, and the excised tissue was sent for histopathological examination, which confirmed ligamentous calcification. Following the complete excision of the calcified SML, the patient's MIO improved to approximately 50 mm, a significant increase from the preoperative measurement of 18 mm.

**Figure 4:**
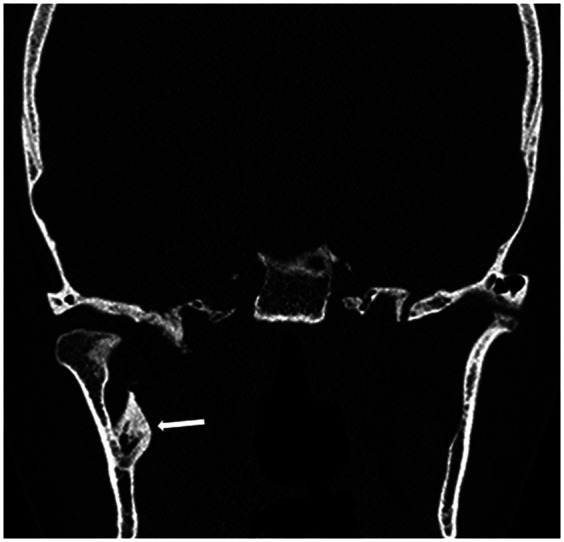
Post-operative coronal plane CT imaging following coronoidectomy and excision of the SML. A small calcification at the lingula was left undisturbed to avoid unnecessary trauma to the surrounding structures (white arrow).

Postoperative imaging, including CT and OPG, was performed to evaluate the complete excision of the ligament. The CT imaging confirmed the successful and complete removal of the calcified SML (Figure 4).

Postoperatively, the patient experienced hypoesthesia in the distribution of the lingual and inferior alveolar nerves, likely due to intraoperative manipulation of the neurovascular bundle. However, sensory function gradually improved over time, with complete resolution of symptoms observed at approximately 3 months posteperatively. The patient underwent intensive physical therapy to further enhance mouth opening and prevent scar formation and contracture.

## Discussion

Calcification of ligaments and soft tissues can result from a variety of factors, including degenerative changes, trauma, metabolic disorders, or idiopathic causes (19–22). In the case of SML, the exact mechanism of calcification remains unclear due to the scarcity of reported cases. However, it is hypothesized that chronic microtrauma or inflammatory processes may contribute to the deposition of calcium salts within the ligament (19–22). The progressive nature of the condition, as seen in the reported cases, suggests that calcification may develop over time, leading to gradual restriction of mandibular movement.

Calcification of the sphenomandibular ligament (SML) is an exceptionally rare phenomenon, with only three documented cases in the medical literature before this report (11, 12). The first case, reported in 2014, involved a 46-year-old male from Bahrain who presented with a painless, progressive limitation in mouth opening, restricted to 12 mm (11). Computed tomography imaging confirmed unilateral calcification of the right SML, and surgical management via intraoral Coronoidectomy resulted in a significant improvement in mouth opening, increasing to 40 mm postoperatively. The second case, reported in 2023, described a 7-year-old male from Yemen with a severe limitation in mouth opening (1 mm) that had persisted since the age of three (12). CT imaging revealed linear radiopacity consistent with calcification of the left SML, and surgical treatment via an intraoral approach with mucoperiosteal flap elevation and ligament dissection yielded successful improvement (11, 12). The third case, reported in 2025, involved an 8-year-old female from Belgium who was referred to the maxillofacial surgery department for evaluation of a rapidly progressive mouth-opening limitation, initially measured at 17 mm. There was no history of medical conditions, surgical procedures, or maxillofacial trauma. CT and MRI confirmed a diagnosis of SML calcification. In this case, surgical intervention was not pursued; instead, the patient underwent maxillofacial physiotherapy, which led to an improvement in mouth opening to 30 mm over a six-month period (15).

The etiology of SML calcification remains poorly understood due to the scarcity of reported cases. In the first two cases, the condition presented with a slow, gradual, and painless progression of trismus, similar to our patient (11, 12). Nevertheless, the third published case (15) involved a more rapid onset of trismus, though the precise duration of symptom progression was not clearly defined. Potential contributing factors include chronic microtrauma, inflammatory processes, or idiopathic calcification of the ligament. The absence of systemic inflammation or metabolic abnormalities in our patient further supports the hypothesis that SML calcification may be a localized phenomenon, possibly related to mechanical stress or degenerative changes in the ligament (19–22). However, while the first case was associated with a dental procedure (11), the second and third case had no identifiable preceding event (12, 15), suggesting that the pathogenesis may be multifactorial.

Notably, in the first case, the calcification was associated with a prior dental procedure involving an inferior alveolar nerve (IAN) block (11). Similarly, our patient reported the onset of trismus following a dental intervention requiring an IAN block injection, suggesting a potential iatrogenic component. Therefore, the calcification observed in our case does not appear to be incidental but rather represents a direct sequela of the preceding dental intervention.

The comparison of reported cases, summarized in Table 1, highlights the multifactorial etiology of SML calcification and suggests that surgical intervention offers more effective resolution. While physiotherapy proved successful in a single case (15), it was largely ineffective in the remaining cases (11, 12), which ultimately required surgery. Given the temporal correlation and clinical similarity, the present case bears strong resemblance to the first documented instance (11), supporting the hypothesis that SML calcification may occur as a delayed consequence of regional anesthesia via IAN block. Further studies are warranted to elucidate the underlying mechanisms and to determine potential preventive measures in dental practice.

**Table 1:** Summary of all SML calcification reported.

Case	Origin	Age	Presentation	Etiology	Management
Salha et al. (11)	Bahrain	47	A 3-year history of progressive trismus	IAN block	Surgery
Alrhabi and Altawili (12)	Yemen	7	A 3-year history of progressive trismus	Idiopathic	Surgery
Kechtban et al. (15)	Belgium	8	rapidly progressive trismus	Idiopathic	Physiotherapy
Current case	Russia	32	A 17-year history of progressive trismus	IAN block	Surgery

Given the rarity of this condition, data on long-term outcomes and recurrence rates are limited. Neither of the previously reported cases had extended follow-up or recurrence monitoring. In our case, we opted for a complete excision of the calcified ligament, sparing only a minimally calcified lingula to avoid unnecessary trauma to surrounding structures. This comprehensive surgical approach aims to minimize the risk of recurrence and future limitations in mandibular function. Postoperative imaging confirmed the complete removal of the calcified SML, and the patient demonstrated significant improvement in mouth opening, with a maximal interincisal opening of 50 mm following surgery.

Ethnically and demographically, there appears to be no clear pattern in cases of SML calcification. The first two cases involved 7 and 46 years old Middle Eastern patients, and the third was 8-years old from Belgium. Our case however, involved a 32-year-old male of Russian-Kazakh origin. This suggests that SML calcification can occur across diverse ethnicities and a wide age range, further emphasizing the need for heightened clinical awareness of this condition in patients presenting with unexplained trismus.

From a surgical perspective, we propose Coronoidectomy as the optimal approach for achieving full exposure and excision of the calcified SML. This technique allows for complete removal of the affected ligament, avoiding the potential limitations of partial dissection and addressing the calcification comprehensively. Our experience underscores the importance of thorough preoperative planning and meticulous surgical technique to optimize outcomes in this rare condition. Additionally, the use of postoperative imaging and histopathological examination is critical to confirm the diagnosis and ensure complete excision of the calcified tissue.

In conclusion, calcification of the SML is a rare but clinically significant cause of trismus that should be considered in the differential diagnosis of patients with progressive, painless limitation in mouth opening, particularly when conventional treatments fail to yield improvement. Early recognition and surgical intervention can lead to significant functional improvement, as demonstrated in our case and the two previously reported cases. Further research and long-term follow-up are needed to better understand the etiology, natural history, and optimal management of this rare condition.

## Data Availability

The original contributions presented in the study are included in the article/Supplementary Material, further inquiries can be directed to the corresponding author.
